# Supplementary material for: Functional gene categories differentiate maize leaf drought-related microbial epiphytic communities
Source: PLoS One. 2020 Sep 18;15(9):e0237493. doi: 10.1371/journal.pone.0237493 (PMC7500591; doi:10.1371/journal.pone.0237493)
Supplement: Attachment — Submitted filename: May 20 reply to reviewer 1 PLOS ONE.docx [file pone.0237493.s003.docx]

May 20, 2020

Reply to reviewer 1 comments. Reviewer requests are in italics, and our replies to reviewer 1 are in plain text.

*Reviewer's Responses to Questions*

***Comments to the Author*** *1. If the authors have adequately addressed your comments raised in a previous round of review and you feel that this manuscript is now acceptable for publication, you may indicate that here to bypass the “Comments to the Author” section, enter your conflict of interest statement in the “Confidential to Editor” section, and submit your "Accept" recommendation.

Reviewer #1: (No Response)

Reviewer #2: All comments have been addressed*

 We provide replies to reviewer 1’s comments below.

6. Review Comments to the Author

Please use the space provided to explain your answers to the questions above. You may also include additional comments for the author, including concerns about dual publication, research ethics, or publication ethics. (Please upload your review as an attachment if it exceeds 20,000 characters)

Reviewer #1: The revised manuscript by Methe et al. makes some clarifications regarding various points, but I think it still lacks enough detail in a number of aspects. I also have some methodological comments.


*1. I am still surprised by the lack of soil sequences. The authors write in their response that methods were specifically designed for leaf epiphytes but there is no clear methodological description of how soil samples were generated. DNA extraction with PowerSoil would certainly produce a high number of microbial DNA from soil samples if standard soil sampling methods were used. Please include a description of the precise soil sampling method.*

Since the goal was to check the extent of soil microbial DNA on leaves due to soil particles on the leaf surface, the soil samples were extracted using the same method that was used for the leaf epiphyte samples (placing soil samples taken adjacent to plants into sterile water with Silwet, filtering, and extracting). We added an additional sentence in the methods to explain this more clearly.

*2. The authors of the HUMaN2 pipeline do state in their website that their method can be used for any community. However, the HUMaN2 publication (doi: 10.1038/s41592-018-0176-y) makes no such claim and only provides analysis for human and marine communities. The methods may be general but since it is a reference based approach, it will always be sensitive to the reference database. Therefore, I urge great caution when interpreting results from this approach on plant-associated communities.*

We added additional explanation of the fact that this is a reference-based approach to the methods section.

*3. Line 190. I had asked about the false discovery rate (FDR) and the author state that they chose the lowest possible false positive rate (FPR) but they never state what was that rate. I think the final FDR/FPR ratio needs to be reported in the manuscript otherwise one doesn’t know how ot interpret the results. Also, FPR and FDR are not identical and the authors should control via FDR not FPR.*

We added additional explanation to the main manuscript, with information about where in the supplemental methods additional information such as the confusion matrices may be found. We created groups (A, B, C) with sequences from the same gene family and different gene families; A and B were low-similarity, while A and C were typical higher similarity levels. When we compared different combinations of groups, AB, BC, AC, the FDRs after running through the ENNB pipeline with TMM normalization were 0.846, 0.195, and 0.088 respectively using the threshold of 0.001 to compare p-values. The goal for the simulations to determine if ENNB was a viable method for detecting gene counts between groups. The low FDR calculated using simulations on groups A and C allowed us to determine that ENNB would be an acceptable tool to run against the real data provided the sequence match value to declare function similarity was set to the higher level. The notebook is genefamilies_simulations.Rmd.

*4. line 220 states that “little correlation between depth of microbial sequence and annotation quality (Table 1).”, but the table doesn’t actually shows that. It just tells me that some samples were sequenced different amounts, but there is no quality metrics.*

We corrected this sentence, thank you for pointing this out.

*5. Lines 295-297 state that there is a correlation between drought severity and similarity in microbial functional changes, but with only three sites and without a statistical analysis to back it, I don’t see how that apparent correlation can be interpreted.*

We agree that comparisons across sites are qualitative, and ‘correlation’ in this sentence is a descriptive term, not a specific measurement.

*6. From the title abstract and conclusion it seems as if as if functional categories consistently differentiate between drought and well-watered conditions; however, in their response the authors state that “The goal of using multiple sites is to illustrate that functional gene changes are likely to be site-specific”. I think the authors need to be more clear about what conclusion they are trying to present. Moreover, claiming site specific differences requires comparisons between sites which the authors refrain from because of lack of appropriate statistical tools. I think this is a valid argument, but then the authors need to be explicit about the limitations of their conclusions. One can make claims about specific sites, but it is impossible to claim if the differences between sites are particularly big or small because we don’t know what to expect.*

We edited the conclusion to clarify this point.

*Reviewer #2: Why the agronomic data found in figure 4 was drawn from different sites? For example a) plant height of the California-Albany (CA) site, b) Seed weight of the Texas-Dumas-Etter site, c) Seed weight of the Texas-Halfway site, d) stem diameter of the California site -Albany ... The same agronomic data record was made for the different sites, because the comparison between the sites in the different agronomic variables analyzed is not illustrated.
Ideally, the effects of affected plants on the effects of plants against drought can be shown, images of plants grown under both conditions, perhaps modify Figure 4 or attach it as supplementary material.
Why use different degrees of drought between different sites and they were not the same at all sites?
How to consider the height of the plants, at what height of the plant to take the diameter of the plant, the weight of the seeds of how many seeds and in the condition raised? It is not described in materials and methods or if the strategy was taken from previous work.
The sizes of the graphics and letters of the axes are small, they need to be edited and the figures must be loaded according to the PLOS ONE guidelines.*

We have uploaded our correctly formatted figure as an eps file.
